# Effect of preoperative nutritional support in malnourished patients with pancreatobiliary cancer: a quasi-experimental study

**DOI:** 10.1186/s40795-022-00555-2

**Published:** 2022-07-11

**Authors:** Hyeong Min Park, Young Hwa Kang, Dong Eun Lee, Mee Joo Kang, Sung-Sik Han, Sang-Jae Park

**Affiliations:** 1 Center for Liver and Pancreatobiliary Cancer, National Cancer Center, Goyang-si, Gyeonggi-do South Korea; 2 Biometric Research Branch, Research Institute and Hospital, National Cancer Center, Goyang-si, Gyeonggi-do South Korea; 3 The Korea Central Cancer Registry, National Cancer Center, Goyang-si, Gyeonggi-do South Korea; 4 Nutrition Support Team, National Cancer Center, Goyang-si, Gyeonggi-do South Korea

**Keywords:** Pancreatobiliary cancer, Preoperative nutritional support program, Malnourished, well-nourished, Postoperative outcome

## Abstract

**Background:**

In malnourished patients, postoperative morbidity, hospitalization period, and medical expenses are reportedly to be high. We evaluated the clinical impact of a preoperative nutritional support program (PNSP) among malnourished cancer patients.

**Methods:**

For this quasi-experimental study, we enrolled 90 patients who underwent major pancreatobiliary cancer surgery. Malnutrition was defined as at least one of the following: (1) Patient-Generated Subjective Global Assessment (PG-SGA) grade B or C; (2) > 10% weight loss within 6 months; (3) body mass index <18.5 kg/m^2^; and (4) serum albumin level < 3.0 g/dL. Forty-five malnourished patients allocated to the PNSP group received in-hospital PNSP for a median of 6 (4–35) days. In the PNSP group, the nutrition support team calculated the patients’ daily nutritional requirements based on their nutritional status and previous day’s intake. The supplementation targets were as follows: total calorie intake, 30–35 kcal/kg/day; protein intake, 1.2–1.5 g/kg/day; and lipid intake, 1–1.5 g/kg/day. Patients who did not meet the diagnostic criteria for malnutrition were allocated to the well-nourished group and underwent surgery without receiving the PNSP (*n* = 45). We compared the perioperative nutritional indices (as measured using PG-SGA), postoperative outcome, and quality of life (QOL) according to the European Organization for Research and Treatment of Cancer Quality of Life Questionnaire version 3.0.

**Results:**

In the PNSP group, the proportion of patients with serum prealbumin <16 mg/dL decreased significantly after PNSP (29.5% vs. 8.9%, *p* = 0.013). Moreover, patients with PG-SGA grade A had a statistically significant increase (2.2% vs. 50%, *p* < 0.001). The overall and major complication rates were higher in the PNSP group than in the well-nourished group without significance (51.1%, 33.3%; 42.2%, 26.7%, respectively). However, the overall and major complication rates were similar between the subgroup with PG-SGA improvement after PNSP and the well-nourished group (40.9% vs. 42.2%, *p* = 0.958; 27.3% vs. 26.7%, *p* = 0.525, respectively). QOL indicators in the PNSP group were comparable with those in the well-nourished group after PNSP.

**Conclusion:**

PNSP may improve perioperative nutritional status and clinical outcomes among malnourished patients with pancreatobiliary cancer.

**Supplementary Information:**

The online version contains supplementary material available at 10.1186/s40795-022-00555-2.

## Background

Although there can be differences depending on the institution or organization, malnutrition is typically defined as a state resulting from lack of nutritional intake leading to altered body composition and body cell mass, causing diminished physical and mental function and impaired clinical outcome from disease [1, 2]. Approximately 20%–40% of hospitalized individuals are assumed to be malnourished [3–5]. This nutritional imbalance worsens during the hospitalization period [6]. The risk of malnutrition in patients with pancreatic cancer is higher than 60%, the highest risk among all cancer types [7]. The efficacy of chemotherapy is reduced in undernourished patients with cancer, leading to need for more treatment breaks and dose reductions of therapy [8, 9]. The postoperative course of these patients leads to increased morbidity and mortality, increased length of hospital stay (LOS), and increased readmission rates, which in turn lead to increased medical expenses [10–14]. According to previous studies, the deaths of 10%–20% of patients with cancer can be attributed to malnutrition rather than to the malignancy itself [15–17].. In addition, several studies have reported that nutritional status affects quality of life (QOL) [18].

In patients who have undergone major surgeries for cancer, malnutrition can increase both morbidity and mortality [19, 20]. These patients also have a prolonged LOS, leading to increased hospital cost and declining QOL. In the clinical setting, however, access to dietary assessment and nutritional support are not available due to limited staffing and resource availability [16, 21]. In 2018, our team reported that the LOS of malnourished patients [Patient-Generated Subjective Global Assessment (PG-SGA) grade B or C] was longer than that of well-nourished patients (PG-SGA grade A). Postoperative complication rates also tended to be higher in malnourished patients than in well-nourished patients [22].

To improve postoperative outcomes, malnutrition must be corrected in patients before undergoing major surgery for cancer. Hence, we hypothesized that providing a preoperative nutritional support program (PNSP) to malnourished patients could lead to comparable clinical outcomes between malnourished and well-nourished patients. However, there are no standardized data on the duration, amount, route, or type of PNSP, particularly in patients with pancreatobiliary cancer who have a significantly higher risk of malnutrition than patients with other cancers including stomach, liver, and colon cancers [22]. On the basis of the European Society for Clinical Nutrition and Metabolism (ESPEN) guidelines, the indications for preoperative nutritional support are as follows: (1) weight loss of >10%–15% within 6 months; (2) body mass index (BMI) of <18.5 kg/m^2^; (3) PG-SGA grade C or nutritional risk screening (NRS) score of >5; and (4) preoperative serum albumin level of <30 g/L (with no evidence of hepatic or renal dysfunction) [23]. The PG-SGA and NRS are validated nutrition assessment tools to detect or screen for malnutrition in patients with cancer [6, 24].

Through prospective enrollment of patients with pancreatobiliary cancer and providing PNSP to the patients who met ESPEN malnutrition criteria, this study aimed to identify the clinical effect of providing PNSP for malnourished patients with pancreatobiliary cancer and to compare the differences between malnourished patients with pancreatobiliary cancer who were given PNSP and well-nourished patients with pancreatobiliary cancer.

The primary endpoint was the overall and major complication rates after surgery. The secondary endpoints were total and postoperative LOS and QOL.

## Methods

### Preoperative nutritional risk screening and group allocation

We prospectively recruited 102 patients with pancreatobiliary cancer who underwent surgery at the National Cancer Center, Korea between October 2014 and October 2016. The inclusion criteria were as follows: (1) patients who underwent a major surgery for pancreatobiliary cancer with curative intent; (2) those aged 20–80 years; (3) those with a good performance status (Eastern Cooperative Oncology Group [ECOG] score of 0 or 1); (4) those with normal primary functions (hemoglobin level: ≥7.0 g/dL, absolute neutrophil count level: ≥1,500/mm3, platelet level: ≥80,000/mm3, aspartate aminotransferase/alanine aminotransferase level ≤5× the normal range, and creatinine level ≤2.0 the upper limit of normal); and (5) those who provided written informed consent. Twelve patients dropped out of the study because of minor or palliative pancreatobiliary surgery (*n* = 7), benign disease in the final pathological result (*n* = 3), or cancellation of surgery (*n* = 2).

**Fig. 1:**
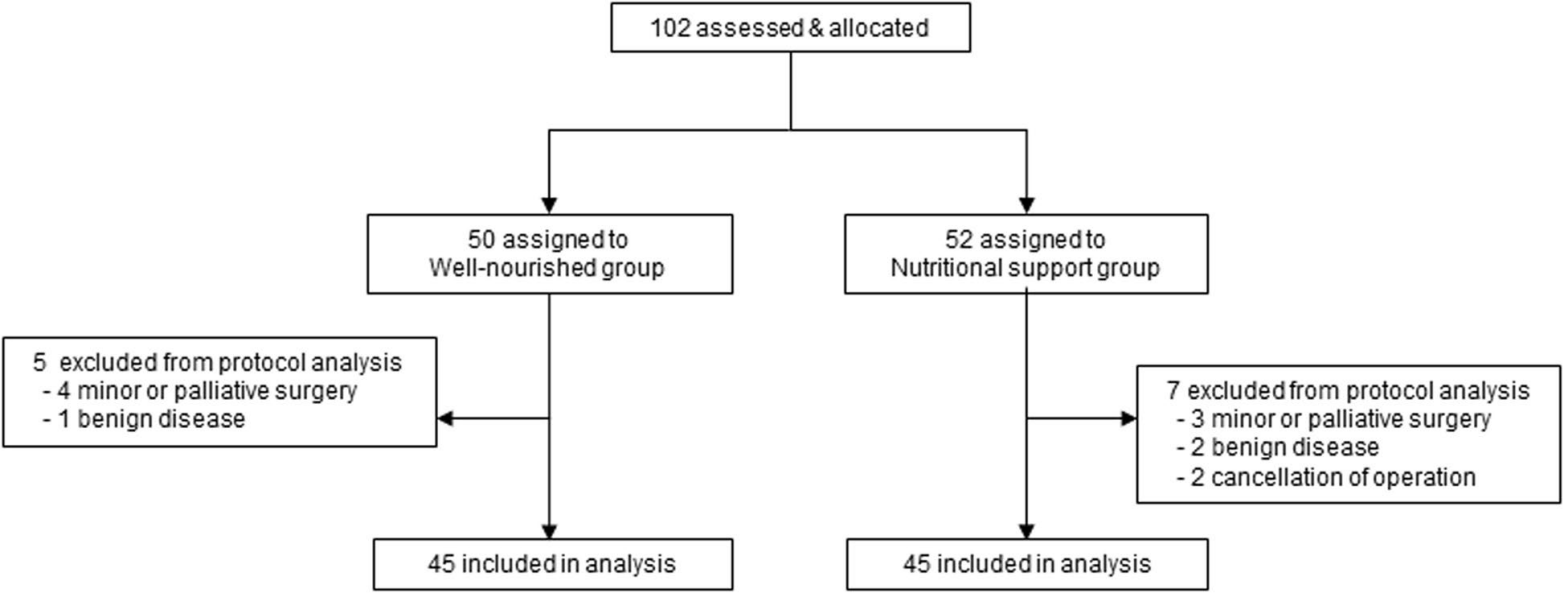
Patient enrolment, assignment, and follow-up

Malnutrition was defined as at least one of the following: (1) PG-SGA grade B or C (*n* = 44); (2) weight loss of >10% within 6 months (*n* = 13); (3) BMI of <18.5 kg/m^2^ (*n* = 3); and (4) serum albumin level of <3.0 g/dL (*n* = 2). On the basis of these diagnostic criteria for malnutrition, the patients enrolled were allocated to the PNSP group or the well-nourished group. All the malnourished patients were enrolled in the PNSP group and underwent surgery after the PNSP for 5─10 days (*n* = 45). Meanwhile, the patients who did not meet the diagnostic criteria for malnutrition were allocated to the well-nourished group and underwent surgery without receiving PNSP (*n* = 45) (Fig. 1). Laboratory examination findings reflected nutritional status, including hemoglobin, total protein, albumin, prealbumin, transferrin, and cholesterol levels. The European Organization for Research and Treatment of Cancer Quality of Life Questionnaire version 3.0 (EORTC QLQ-C30) [25–27] was used for evaluating the patients’ QOL. Trial approval was obtained from the institutional ethics committee (Institutional Review Board, National Cancer Center, Korea) before starting the study (NCCCT13676). This study was registered on ClinicalTrials.gov (NCT02626195).

### Preoperative nutritional support program

During the study period, patients assigned to the PNSP group were provided preoperative nutrition supplementation with the target of 5–10 days during their hospital course. The clinical staff composed of doctors, dietitians, and nutrition support nurses, calculated and provided the patients’ daily energy and protein requirements during the PNSP period based on the patients’ nutritional status, the route of nutritional support, and the previous day’s intake. The supplementation targets were as follows: total calories 30–35 kcal/kg/day; protein 1.2–1.5 g/kg/day; and lipids 1–1.5 g/kg/day. Moreover, various minerals and vitamins were also provided. If oral intake was insufficient (<80% of the caloric target), an oral high-calorie supplement (Encover®, JW Pharmaceutical Co., Ltd., Korea) was provided (Table S1). For patients who did not reach 60% of the caloric target, supplementary parenteral nutritional support was administered (Smofkabiven®^,^ Fresenius Kabi Korea Ltd., Korea) to achieve >80% of the target caloric, protein, and lipid intake. In principle, PNSP was applied until the day before surgery, and dextrose and crystalloid fluids were supplied after midnight of the day before surgery. Oral fasting was limited to 3 h for liquids and 8 h for solids. Prior to surgery, the nutritional and performance status indicators (BMI and ECOG score), laboratory parameters (total protein, albumin, prealbumin, transferrin, and cholesterol levels), PG-SGA grade and score, and QOL as measured using the EORTC QLQ-C30 score of the PNSP group were evaluated. The PNSP group then underwent surgery within 3 days after receiving nutritional support.

### Postoperative nutritional support and postoperative nutritional risk screening

The nasogastric tube was removed if the output fell below 500 mL/day and if no gastric distension or air-fluid level in the stomach was observed on chest X-ray on postoperative day (POD) 1. Sips of water were initiated on POD 2, a soft fluid diet on POD 4, a soft blended diet on POD 6, and a normal regular diet on POD 13. Postoperative nutritional support was provided to both groups based on our enhanced recovery programs, which include an early and step-by-step gradual increase in oral intake combined with supplementary partial parenteral nutrition (PN). The postoperative target caloric intake was 25–30 kcal/kg/day. The postoperative target protein and lipid intake were the same as those of the PNSP. On POD 14, the physical status, complete blood count, blood chemistry level, EORTC QLQ-C30 score, and PG-SGA score and grade were evaluated. Two weeks after discharge, the same factors were assessed, except for the EORTC QLQ-C30 score and the PG-SGA score and grade. The medical record, including the hospital course, was reviewed by the attending physicians and dietitians after discharge to validate all information on the data collection checklist. The postoperative complications were graded using the Clavien–Dindo classification system [28]. The LOS was classified as whole and postoperative LOS. Whole LOS was defined as the duration from the day of hospitalization to the day of discharge, and postoperative LOS as the time between day of surgery and day of discharge.

### Prospective study protocol

In a previous analysis of data on outcomes after surgery in patients with pancreatobiliary malignancy in this institution (NCCNCS-11-460), malnourished patients showed a higher postoperative complication rate than well-nourished patients, although there was no significant difference (45% vs. 36%, *p* = 0.4). Moreover, the major postoperative complication rate (Clavien–Dindo classification score of ≥3) in malnourished patients was significantly higher than that in well-nourished patients (25% vs. 8%, *p* = 0.03). Additionally, the LOS in the hospital of the PNSP group was longer than that of the well-nourished group (20 vs. 14 days, *p* = 0.013). On the basis of our previous experience, we hypothesized that the major complication rate of malnourished patients could be reduced from 25% to 10% after PNSP. To achieve a power of 85% considering a 10% follow-up loss, 50 participants were needed in each group for a one-sided test with a type I error rate of 5%.

In this quasi-experimental study, we included everyone who met the inclusion criteria and provided informed consent to the study. All patients admitted to this study were screened consecutively for eligibility to avoid selection bias. To minimize performance and detection bias, neither of preoperative nor postoperative data, were analyzed before the study was completed.

### Statistical analysis

Several factors associated with nutritional status and postoperative outcomes were analyzed to compare the difference between the PNSP and well-nourished groups. Additionally, to identify the effect of PNSP in malnourished patients, a subgroup analysis was performed by dividing the PNSP group to two subgroups according to whether the nutritional status was improved or not (PG-SGA grade B/C → B/C subgroup and PG-SGA grade B/C → A subgroup). The factors analyzed were presented as numbers and percentages for categorical variables. Pearson’s chi-squared test or Fisher’s exact test was used to compare the categorical variables between the groups. Differences between the continuous variables were analyzed with the Wilcoxon signed-rank test or the Wilcoxon rank sum test. A regression model was performed to identify the risk factor for malnutrition. A *p*-value of <0.05 was considered statistically significant. The collected data were analyzed with IBM SPSS version 22.0 (IBM Corp., Armonk, NY, USA).

## Results

### Patients’ baseline characteristics

The ECOG score (37.8% vs. 17.8%, *p* = 0.034), elevated serum carbohydrate Antigen 19-9 (CA 19-9) levels (70.5% vs. 34.9%, *p* = 0.001), and the rate of poorly differentiated cancer (45.0% vs. 21.2%, *p* = 0.047) were significantly higher in the PNSP group than in the well-nourished group. Apart from these factors, there was no significant difference in the baseline characteristics between the PNSP and well-nourished groups (Table 1).

**Table 1:** Baseline characteristics of the two groups

Factors		PNSP	Well-nourished	*p*
		(*n* = 45)	(*n* = 45)	
		n (%) or median (min─max)		
Age	>75 years	7 (15.6%)	4 (8.9%)	0.334*
Sex	Male	30 (66.7)	29 (64.4)	0.824*
	Female	15 (33.3)	16 (35.6)	
ECOG	0	28 (62.2)	37 (82.2)	0.034
	1	17 (37.8)	8 (17.8)	
ASA score	1	4 (8.9)	10 (22.2)	0.071
	2	36 (80.0)	34 (75.6)	
	3	5 (11.1)	1 (2.2)	
Diagnosis	Bile duct cancer	28 (62.2)	22 (48.9)	0.443^#^
	Gallbladder cancer	2 (4.4)	2 (4.4)	
	Pancreatic cancer	15 (33.3)	20 (44.4)	
	Others	0 (0.0)	1 (2.2)	
External biliary drain	No	31 (68.9)	37 (82.2)	0.220^#^
	Yes	14 (31.1)	8 (17.8)	
HbA1c	≥ 6.5%	15 (33.3)	16 (35.6)	0.999^#^
CA 19-9	> 37.0U/mL	31 (70.5)	15 (34.9)	0.001^#^
CEA	> 5.0ng/mL	12 (26.7)	7 (17.1)	0.311^#^
T stage	1 or 2	18 (40.0)	17 (37.8)	0.999^#^
	3 or 4	27 (60.0)	28 (62.2)	
LN metastasis	No	21 (47.7)	18 (40.0)	0.525^#^
	Yes	23 (52.3)	27 (60.0)	
Differentiation	Well or Moderate	26 (78.8)	22 (50.0)	0.047^#^
	Poor	7 (21.2)	18 (45.0)	
Cancer Stage	1 or 2	40 (88.9)	39 (86.7)	0.999
(AJCC 8th)	3 or 4	5 (11.1)	6 (13.3)	
Type of surgery	PD/PPPD	30 (66.7)	27 (60.0)	0.377^#^
	HPD	4 (8.9)	1 (2.2)	
	Hepatectomy + BDR	8 (17.8)	11 (24.4)	
	Distal panceatectomy	3 (6.7)	6 (13.3)	

*PNSP* preoperative nutritional support program*, ECOG* Eastern Cooperative Oncology Group, *ASA* american society of anesthesiologists, *HbA1c* hemoglobin A1c, *CA 19-9* Carbohydrate Antigen 19-9, *CEA* carcinoembryonic antigen, *LN* Lymph node, *PD* pancreatoduodenectomy, *PPPD* pylorus preserving pancreatoduodenectomy, *HPD* hepaticopancreatoduodenectomy, *BDR* bile duct resection

*chi-square test, #Fisher’s exact test

In the regression analysis to identify the risk factors for preoperative malnutrition in patients with pancreatobiliary cancer, ECOG score, ASA score, preoperative serum CA 19-9 level, and cell differentiation showed significance in the univariate analysis. Among these factors, elevated serum CA 19-9 level was an independent risk factor for preoperative malnutrition in patients with pancreatobiliary cancer (Table S2).

However, the baseline characteristics between the PG-SGA grade B/C → A (*n* = 22) and PG-SGA grade B/C → B/C subgroups (*n* = 23) even after receiving nutritional support did not differ (Table S3).

### Efficacy of the preoperative nutritional support program

The patients in the PNSP group received the PNSP for 4─35 (median: 6) days. In total, 42 (93.3%) patients in the PNSP group received PNSP for between 5 and 10 days. The median value of the caloric intake achieved was 32.1 (range: 20.3─44.5) kcal/kg/day, and the median protein intake achieved was 1.3 (range: 0.8─2.0) g/kg/day. The median lipid intake was 1.0 (range: 0.3─1.7) g/kg/day. In total, 11 (24.4%) patients received supplementation with oral intake; 20 (44.4%) received oral intake with an oral supplement; and 14 (31.1%) received oral intake and an oral supplement with PN. All the patients in the PNSP group achieved up to ≥80% of the target calories, and 90% were supplied with up to ≥80% of the target protein intake. In total, 14 patients received PN for 4 (interquartile range [IQR] 1.5–5) days, with a median caloric support of 483 (IQR 361.2─940.3) kcal/day. The duration of nutritional support in three patients was not within the recommended time (5–10 days). A 72-year-old male patient who received preoperative nutritional support for only 4 days underwent extended right hemi-hepatectomy with bile duct resection for Klatskin tumor at surgeon’s discretion. A 71-year-old male patient who received nutritional support for 35 days had several percutaneous transhepatic biliary drains for obstructive jaundice due to Klatskin tumor. The PNSP duration was extended to 17 days in a 74-year-old woman with pancreatic cancer due to acute cholangitis before the Whipple procedure.

The outcomes of the PNSP between the PG-SGA grade B/C → B/C and PG-SGA grade B/C → A subgroups differed in terms of the protein intake (g/kg/day) achieved (1.2 vs. 1.4, *p* = 0.005) and the percentage of successful days of target protein intake within the total PNSP days (69% vs. 100%; *p* = 0.001). The rate of successful days of target caloric intake within the total PNSP days differed between the two subgroups. The difference had a marginal significance (94% vs. 100%, *p* = 0.051). However, there was no significant difference in terms of nutritional indices after PNSP between the two subgroups (Table S3). Daily checks by the nutritional support team and the clinicians by reviewing the electronic medical records showed no adverse events related to PNSP.

### Nutritional status after preoperative nutritional support, POD 14, and 2 weeks after discharge

Patients who received PNSP had significant improvements in prealbumin levels and PG-SGA grades and scores. However, despite receiving nutritional support, the proportion of patients with anemia and low transferrin, as well as PG-SGA grade and score, in the PNSP group did not reach that of the well-nourished group (Table 2).

**Table 2:** Preoperative nutrition indicators

Variables		PNSP (*n* = 45)					
		Before Support	After Support	*p*^‡^	Well-nourished (*n* = 45) n (%)	(vs Before support) ^†^	*p* (vs After support) ^†^
		n (%)					
BMI < 18.5 kg/m^2^		3 (6.7%)	2 (4.4%)	0.645	0	0.078	0.078
ECOG	0	28 (62.2%)	25 (55.6%)	0.458^#^	37 (82.2%)	0.034	0.020
	1	17 (37.8%)	19 (42.2%)		8 (17.8%)		
Anemia M<13/F<12g/dL		30 (66.7%)	36 (80.0%)	0.233^#^	11 (24.4%)	<.001^#^	< .001^#^
Albumin level < 3 g/dL		2 (4.4%)	1 (2.2%)	0.557	0	0.153	0.315
Protein level < 6 g/dL		10 (22.2%)	8 (17.8%)	0.598	3 (6.7%)	0.036	0.108
Cholesterol > 200 mg/dL		10 (22.2%)	7 (15.6%)	0.419	10 (22.2%)	0.999	0.419
Prealbumin < 16 mg/dL		13 (29.5%)	4 (8.9%)	0.013	4 (8.9%)	0.013	>.999
Transferrin <170 mg/dL		6 (13.6%)	7 (15.6%)	0.798	1 (2.2%)	0.046	0.026
PG-SGA grade	A	1 (2.2)	22 (50.0)	<.001^§^	45 (100.0)	<.001	< .001^#^
	B	41 (91.1)	21 (47.7)				
	C	3 (6.7)	1 (2.3)				
PG-SGA score		11.0 (4.0-20.0)	6.0 (2.0-20.0)	<.001^†^	4.0 (1-8.0)	<.001^†^	< .001^†^

*PNSP* preoperative nutritional support program, *ECOG* Eastern Cooperative Oncology Group *PG-SGA* Patient-Generated Subjective Global Assessment

Two weeks after the surgery, there was no significant difference between the two groups, except for the proportion of patients with low transferrin levels (72.5% vs. 47.7%, *p* = 0.021) (Table 3).

**Table 3:** Analysis of nutritional status at postoperative day 14 and 2 week after discharge

Variables		PNSP	Well-nourished	*p*
		(*n* = 43)	(*n* = 45)	
		N (%)	N (%)	
Postoperative day 14				
BMI <18.5kg/m2		2 (4.7%)	1 (2.2%)	0.53
Anemia		39 (92.9%)	42 (93.3%)	0.93
ECOG	0	4 (9.3%)	11 (24.4%)	0.389
	1	35 (81.4%)	33 (73.3%)	
	2	4 (9.3%)	1 (2.2%)	
Albumin <3g/dL		8 (19.0%)	7 (15.6%)	0.667
Protein <6g/dL		21 (50.0%)	29 (64.4%)	0.173
Cholesterol >200mg/dL		0	2 (4.4%)	0.167
Prealbumin<16mg/dL		35 (87.5%)	37 (84.1%)	0.656
Transferrin<170mg/dL		29 (72.5%)	21 (47.7%)	0.021
Two weeks after discharge				
BMI <18.5kg/m2		6 (14.3%)	4 (9.1%)	0.453
Anemia		39 (92.9%)	42 (93.3%)	0.93
ECOG	0	15 (35.7%)	20 (45.5%)	0.47
	1	26 (61.9%)	21 (47.7%)	
	2	1 (2.4%)	3 (6.8%)	
Albumin <3g/dL		0	0	
Protein <6g/dL		1 (2.4%)	4 (9.1%)	0.184
Cholesterol >200mg/dL		2 (4.8%)	3 (6.8%)	0.684
Prealbumin<16mg/dL		8 (19.0%)	12 (27.3%)	0.367
Transferrin<170mg/dL		6 (14.3%)	5 (11.4%)	0.685
PG-SGA grade	A	12 (31.6)	17 (39.5)	0.498^#^
	B	23 (60.5)	25 (58.1)	
	C	3 (7.9)	1 (2.3)	
PG-SGA score		10.0 (1.0-21.0)	9 (1.0-22.0)	0.275

*PNSP* preoperative nutritional support program, *BMI* Body Mass Index*, ECOG* Eastern Cooperative Oncology Group

^#^Fisher’s exact test

### Subgroup analysis for nutritional status after POD 14, and 2 weeks after discharge

In the subgroup analysis, the proportion of patients with transferrin deficiency was significantly higher in the PG-SGA grade B/C → B/C subgroup than in the PG-SGA grade B/C → A subgroup and well-nourished group. However, the proportion of patients with transferrin deficiency was comparable between the PG-SGA B/C → A subgroup and well-nourished group. (Table S4). Two weeks after discharge, the PG-SGA grade and score were comparable between the two PNSP subgroups and the well-nourished group. They were also comparable between the two PNSP subgroups. The proportion of patients with transferrin deficiency decreased in both subgroups. However, it was still higher in the PG-SGA grade B/C → B/C subgroup than in the other groups (Table S4).

### Clinical outcomes of preoperative nutritional support program

The overall and major complication (Clavien–Dindo classification score of ≥ 3) rates were higher in the PNSP group (51.1% and 33.3%, respectively) than in the well-nourished group (42.2% and 26.7%, respectively), but the difference did not reach statistical significance The median postoperative LOS in the hospital did not significantly differ between the PNSP and the well-nourished groups (17 and 15 days, respectively). During the study period, four patients (three in the PNSP group and one in the well-nourished group) died because of postoperative complications (bleeding, *n* = 2; sepsis, *n* = 2).

### Subgroup analysis for clinical outcomes of preoperative nutritional support program

In a subgroup analysis according to the improvement in nutritional status after PNSP, the overall complication rate was not significantly different among the PG-SGA grade B/C → B/C subgroup (*n* = 21), PG-SGA grade B/C → A subgroup (*n* = 22), and well-nourished group (*n* = 45) (61.9%, 40.9%, and 42.2%, respectively). Although the results did not significantly differ, the major postoperative complication rate was higher in the PG-SGA B/C → B/C subgroup than in the PG-SGA B/C → A subgroup and well-nourished group (42.9%, 27.3%, and 26.7%, respectively). The rate of perioperative red blood cell (RBC) transfusions in the PG-SGA grade B/C → B/C subgroup was significantly higher than that in the well-nourished group (33.3% vs. 8.9%; *p* = 0.022). However, there was no significant difference in terms of the rate of perioperative RBC transfusion between the PG-SGA grade B/C → A subgroup and well-nourished group (18.2% vs. 8.9%; *p* = 0.271). The postoperative LOS in the hospital of the PG-SGA grade B/C ➔ B/C subgroup was longer than that of the PG-SGA grade B/C → A subgroup (21 vs. 16 days, *p* = 0.146). However, the result was not significantly different. The postoperative LOS in the hospital was similar between the PG-SGA B/C → A subgroup and well-nourished group (16 vs. 15 days, *p* = 0.957) (Table 4).

**Table 4:** Comparison of postoperative outcomes between the PNSP and well-nourished groups. Subgroup analysis according to whether nutritional status improved

Variables	PNSP (n = 43)		Well-nourished (n = 45)			
	B/C -> B/C(n=21)		B/C -> A (n=22)			
	n (%) or median (min-max)		*p*		*p* (vs. B/C->B/C)	*p* (vs. B/C->A)
Op. time (min)	345 (215-625)	323 (95-700)	0.173†	325 (140-645)	0.127†	0.785†
EBL (mL)	550 (0-3900)	400 (100-3500)	0.327†	400 (0-2200)	0.056†	0.614†
Complications	13 (61.9)	9 (40.9)	0.169	19 (42.2)	0.194	0.918
Major complications	9 (42.9)	6 (27.3)	0.284	12 (26.7)	0.238	0.958
CR-POPF	3 (13.0%)	1 (4.5%)	0.317	4 (8.9%)	0.594	0.525
Postop. bleeding	3 (13.0%)	3 (13.6%)	0.953	5 (11.1%)	0.815	0.765
RBC transfusion	7 (33.3)	4 (18.2)	0.310^#^	4 (8.9%)	0.022	0.271
LOS (whole)	34 (14-63)	25 (12-83)	0.310†	20 (10-65)	<.001	0.050†
LOS (postop.)	21 (7-57)	16 (3-75)	0.240†	15 (8-63)	0.146	0.957†

*PNSP* preoperative nutritional support program, *EBL* estimated blood loss, *CR-POPF* clinical relevant postoperative pancreatic fistula, *RBC* red blood cell, *LOS* length of hospital stay

### Comparison of QOL before and after nutritional support

The QOL evaluation of six patients before PNSP and one patient after PNSP in the PNSP group could not be performed because of noncompliance. The QOL on POD #14 of five patients in the PNSP group could not be evaluated because of decline evaluation (*n* = 2) or postoperative death of the patient (*n* = 3). In the well-nourished group, the evaluation of QOL on POD #14 could not be obtained for two patients (refusal by one patient and loss of questionnaire of another patient).

After PNSP, several factors associated with QOL including functional scale scores, role functioning, emotional functioning, symptom scale scores, fatigue, pain, and loss of appetite improved in the PNSP group. However, even after receiving PNSP, the nutritional support and well-nourished groups still differed in terms of global health status, physical functioning, and appetite loss (Table 5). However, all factors in the EORTC QLQ-C30 were comparable between the nutritional support and well-nourished groups at POD 14 (Table 6).

**Table 5:** Preoperative EORTC QLQ-C30 scores of the participants

Dimension	PNSP		*p*^‡^	Nutritional support	Well-nourished	*p*^†^
	Before	After NSP		After NSP		
	(*n*= 39)	(n= 39)		(*n*= 44)	(n= 45)	
	median (min─max)				median (minm─max)	
Global health status QOL						
Global health status/QOL	41.7 (0.0─100.0)	50.0 (0.0 - 100.0)	0.258	50.0 (0.0─100.0)	50.0 (0.0─91.7)	0.020
Functional scales	75.6 (33.3─100.0)	82.2 (46.7 - 1100.0)	0.024	83.3 (28.9─100.0)	84.4 (22.2─100.0)	0.755
Physical functioning	80.0 (26.7─100.0)	80.0 (33.3 - 100.0)	0.334	80.0 (20.0─100.0)	86.7 (33.3─100.0)	0.016
Role functioning	66.7 (0.0─100.0)	83.3 (16.7 - 100.0)	0.016	83.3 (0.0─100.0)	100.0 (0.0─100.0)	0.192
Emotional functioning	75 (41.7─100.0	91.7 (50.0 - 100.0	<.001	91.7 (16.7─100.0)	83.3 (8.3─100.0)	0.209
Cognitive functioning	83.3 (0.0─100.0)	83.3 (33.3 - 100.0)	0.222	83.3 (33.3─100.0)	83.3 (16.7─100.0)	0.429
Social functioning	66.7 (0.0─100.0)	83.3 (0.0 - 100.0)	0.500	83.3 (0.0─100.0)	66.7 (0.0─100.0)	0.441
Symptom scales	27.3 (0.0─75.8)	18.2 (0.0 - 72.7)	0.001	19.1 (0.0─72.7)	18.2 (0.0─60.6)	0.211
Fatigue	44.4 (0.0─100.0)	33.3 (0.0 - 100.0)	0.001	33.3 (0.0─100.0)	22.2 (0.0─88.9)	0.166
Nausea and vomiting	0.0 (0.0─100.0)	0.0 (0.0 - 66.7)	0.150	0.0 (0.0─66.7)	0.0 (0.0─66.7)	0.230
Pain	33.3 (0.0─83.3)	0.0 (0.0 - 100.0)	0.019	0.0 (0.0─100.0)	16.7 (0.0─100.0)	0.662
Dyspnea	0.0 (0.0─66.7)	0.0 (0.0 - 66.7)	0.437	0.0 (0.0─66.7)	0.0 (0.0─33.3)	0.975
Insomnia	33.3 (0.0─100.0)	0.0 (0.0 - 66.7)	0.054	0.0 (0.0─100.0)	0.0 (0.0─66.7)	0.595
Appetite loss	66.7 (0.0-─100.0)	33.3 (0.0 - 100.0)	0.002	33.3 (0─100.0)	0.0 (0.0─100.0)	0.005
Constipation	0.0 (0.0─100.0)	0.0 (0.0 - 100.0)	0.492	0.0 (0.0─100.0)	0.0 (0.0─100.0)	0.238
Diarrhea	0.0 (0.0─100.0)	0.0 (0.0 - 100.0)	0.597	0.0 (0.0─100.0)	0.0 (0.0─100.0)	0.682
Fnancial difficulties	33.3 (0.0─100.0)	33.3 (0.0 - 100.0)	0.171	0.0 (0.0─100.0)	33.3 (0.0─100.0)	0.720

*PNSP* preoperative nutritional support program, *NSP* nutritional support program, *QOL* quality of life

^‡^Wilcoxon signed rank test, ^†^Wilcoxon rank sum test, high score for a functional scale : healthy level of functioning, high score for the global health status : high QoL, high score for a symptom scale

**Table 6:** Postoperative EORTC-30 scores of the participants

Dimension	PNSP	Well-nourished	*p*^†^
	(n = 40)	(n = 43)	
	median (min─max)		
Global health status/QOL	50.0 (0.0─100.0)	50.0 (0.0─100.0)	0.854
Functional scales	73.3 (11.1─95.6)	68.9 (40.0─100.0)	0.672
Physical functioning	60.0 (0.0─93.3)	73.3 (33.3─100.0)	0.056
Role functioning	66.7 (0.0─100.0)	66.7 (0.0─100.0)	0.495
Emotional functioning	79.2 (8.3─100.0)	75.0 (33.3─100.0)	0.922
Cognitive functioning	75.0 (0.0─100.0)	66.7 (16.7─100.0)	0.970
Social functioning	66.7 (0.0─100.0)	66.7 (0.0─100.0)	0.714
Symptom scales	34.8 (0.0─78.8)	33.3 (6.1─57.6)	0.491
Fatigue	44.4 (0.0─100.0)	44.4 (0.0─77.8)	0.602
Nausea and vomiting	16.7 (0.0─83.3)	0.0 (0.0─66.7)	0.344
Pain	33.3 (0.0─100.0)	33.3 (0.0─100.0)	0.516
Dyspnea	33.3 (0.0─100.0)	0.0 (0.0─100.0)	0.053
nsomnia	33.3 (0.0─100.0)	33.3 (0.0─100.0)	0.931
Appetite loss	66.7 (0.0─100.0)	33.3 (0.0─100.0)	0.505
Constipation	0.0 (0.0─100.0)	33.3 (0.0─100.0)	0.774
Diarrhea	0.0 (0.0─100.0)	0.0 (0.0─100.0)	0.456
Financial difficulties	33.3 (0.0─100.0)	33.3 (0.0─100.0)	0.679

^†^Wilcoxon rank sum test high score for a functional scale : healthy level of functioning, high score for the global health status : high QoL, high score for a symptom scale: problems

## Discussion

Previous studies proposed that the biological pathways and the host-inflammatory response could be more intense in pancreatobiliary cancer than in other malignancies, leading to the exceptionally high malnutrition rate in patients with pancreatic cancer [29]. The biological mechanisms of pancreatobiliary cancer-related malnutrition are multimodal processes, including catabolic effects derived from the inflammatory state and energy and nutritional losses. These include biliary obstruction, exocrine pancreatic insufficiency, anorexia, and poor oral intake derived from anatomical changes due to cancer and the adverse effects of surgical and medical treatments [17, 30, 31]..

CA 19-9, the tumor marker of pancreatobiliary cancer, has been reported as a prognostic factor. A few previous studies have reported that an elevated CA 19-9 level was associated with malnutrition in patients with cancer [32, 33]. Our study also showed that elevated CA 19-9 was more frequent in the PNSP group. These results show that malnutrition is associated with aggressive cancer in patients with pancreatobiliary cancer. However, malnourished patients’ nutritional status improvement was not associated with cancer characteristics, such as the serum tumor marker level, tumor stage, or cell differentiation. In other words, it shows that even in cancer patients with poor characteristics, preoperative nutritional support can improve the patients’ nutritional status.

ESPEN guidelines on nutrition have been published, on the basis of several studies of nutritional support in malnourished patients with cancer [23, 34]. In 2018, the International Study Group on Pancreatic Surgery published a guideline for nutritional support in pancreatic surgery [35]. These guidelines recommended routine assessment for malnutrition prior to major surgery and preoperative nutritional support in malnourished patients. In these guidelines, however, references for preoperative nutritional support are limited, and most are retrospective studies or based on non-pancreatobiliary cancer like colon or lung cancer. Therefore, there are no standardized data on the duration, amount, route, or type of PNSP. To our knowledge, this is the first study to compare the postoperative short-term outcomes between malnourished patients who received PNSP and well-nourished patients who underwent a major operation for pancreatobiliary malignancy.

Previous research using the PG-SGA (a nutritional assessment tool that scores and assigns grades to severity of malnutrition) demonstrated that 47.6% of all patients with pancreatobiliary cancer were malnourished. In the surgery group, the malnutrition rates were high in patients with esophageal cancer (36%) or pancreatobiliary cancer (37%), and the postoperative complication rates were high in these patient populations. Malnourished patients who were older and had a lower BMI had significantly longer LOS in the hospital than well-nourished patients [22].

Complicated surgical interventions such as pancreatobiliary surgery produce intense metabolic and nutritional status changes by activating an inflammatory response and releasing stress hormones and cytokines. Postoperative appropriate tissue healing and organ function recovery can lead to an effective metabolic response if adequate nutrition support is provided [35].

Malnutrition can cause ongoing energy deficits in the postoperative period, increasing the risk of postoperative complications and poorer clinical outcomes [36]. The association between preoperative malnutrition and poor clinical outcome indicates that malnourished patients who undergo surgery might benefit from preoperative nutritional support [37, 38]. Bozzetti et al. showed that preoperative total parenteral nutrition was correlated with a lower rate of major postoperative complications, including infections, in malnourished patients with gastrointestinal cancer [11, 39].

The ESPEN guidelines shows that the appropriate period of preoperative nutritional therapy in patients with severe nutritional risk could be 7─14 days. In this study, 42 (93%) patients in the PNSP group received preoperative nutritional support for 5─10 days. The duration of nutritional support was shorter in this study than in previous ones [23, 40]. However, practical aspects including decreased hospital turnover rates and increased length of waiting time associated with in-hospital PNSP, were taken into consideration in this study. Moreover, delaying the surgery for more than 1 week in patients with pancreatobiliary cancer was a burden to both patients and surgeons. To overcome time limitations for preoperative nutritional support in patients with pancreatobiliary cancer, the patients’ nutritional status and their intake were evaluated daily to adjust the nutritional supplement plan for the following day. Additionally, to achieve target nutrition over a short period, we judged that applying PN would be more effective and lead to better patient compliance than enteral nutrition support. Consequently, the amount of preoperative nutritional supply for all patients reached the target amount with minimal time lags. Hence, the PNSP group experienced improvement in various nutritional indices. Moreover, after receiving PNSP, the nutritional support and well-nourished groups were comparable in various factors, especially between the PG-SGA B/C → A subgroup and well-nourished group. Previous studies have reported that perioperative nutritional support is correlated with lower postoperative complication rates. Similarly, our study showed that the rate of postoperative complications in the PNSP group, particularly in the subgroup with better nutritional status after receiving nutritional support, improved and was comparable with that of the PNSP group.

QOL is of major importance in pancreatobiliary disease; therefore, improvements in QOL variables, in addition to nutritional indices, indicate that the effect of preoperative nutritional support on postoperative outcomes in patients who underwent major pancreatobiliary surgery should not be disregarded. Kim et al. reported that the preoperative QOL score as assessed using the EORTC QLQ-C30 was significantly lower in the malnourished group than in the well-nourished group. In that study, the significance of the QOL difference between the two groups disappeared 1 year after surgery [41]. In the present study, although the two groups were still different in terms of some factors representing QOL after the PNSP, all factors became comparable at POD 14. The improvement in several factors derived from the QOL analysis in the PNSP arm indicates that malnourished patients were able to return to their baseline performance status more quickly than those without this intervention.

Moreover, our study showed that the duration of hospital stay between malnourished patients with better nutritional status after receiving preoperative nutritional support (PG-SGA grade B/C → A) and well-nourished patients was similar. This finding indicates that the correction of preoperative malnutrition is associated with rapid recovery after a major surgery.

Despite the provision of the PNSP, the proportion of patients with anemia in the malnourished group with pancreatobiliary cancer increased. The RBC transfusion rate did not differ between the PG-SGA grade B/C → A subgroup and well-nourished group. However, the RBC transfusion rate was significantly higher in the PG-SGA grade B/C → B/C subgroup than in the well-nourished group. Blood transfusion affects overall survival after major surgeries, and is associated with preoperative hemoglobin levels [42]. Therefore, a strategy is required to improve hemoglobin levels during preoperative nutritional support.

We should focus on malnourished patients with poor improvement in nutritional status even after receiving nutritional support. Short-term PNSP can be associated with insufficient clinical improvement in the PNSP group compared with the well-nourished group. In this study, there were a higher proportion of patients with poor performance status and anemia during PNSP. This finding indicates that increasing the nutritional support period alone is not helpful in improving outcomes. Additionally, factors and strategies in patients who did not experience improvement in nutritional status despite receiving nutritional support should be investigated. The amount of protein intake (g/kg/day) and the rate of successful days that the target protein intake was achieved during PNSP were significantly associated with improvements in nutritional status in malnourished patients with pancreatobiliary cancer. Poor protein supply was caused by the time it took to reduce the difference between the target amount of protein as calculated by the nutrition team and using the actual patient's intake. Yeh et al. reported that high caloric and protein deficits among adult surgical intensive care unit patients receiving enteral nutrition for >72 h made them less likely to be discharged home [43]. Moreover, in patients with pancreatobiliary cancer, malnutrition is caused by multimodal biologic mechanisms. These mechanisms include catabolic conditions leading to nutritional losses, reduced ability to decompose and absorb nutrients caused by biliary obstruction and exocrine pancreatic insufficiency, and anorexia and poor oral intake derived from anatomical changes [17, 30, 31]. Considering these points, it is possible that our PNSP, which approached the patient's nutrition step by step for a short period, from oral diet to venous nutrition, could not meet the actual nutritional requirements for patients with pancreatobiliary cancer. Therefore, a sufficient amount of protein should be provided to malnourished patients within a short period to improve postoperative outcomes. Flexible strategies such as supplying protein with the goal of achieving the upper limit of the expected requirement or by early management with PN are required.

This study had several limitations. It was a single-center study with a small number of patients, indicative of the relatively small sample size of overall patients with resectable pancreatobiliary cancer. Therefore, there was no choice but to conduct a case-control study, instead of a randomized controlled trial. Moreover, there was a limitation in assessing the effect of differences in this small-scale study, and performing a statistical analysis was challenging. Hence, multicenter randomized trials must be performed to overcome the limitations of small-scale studies such as this one. Another limitation is the heterogeneous PNSP period. The target period of the PNSP in this study was 5 days. However, if the surgical schedule was postponed or a holiday continued at the end of the target nutrition period, the PNSP period was further extended according to the surgical schedule. Therefore, it might be limited to confirming the short-term PNSP application's effectiveness. Additionaly, given that the major surgery for pancreatobiliary cancer is a complex procedure and pancreatobiliary cancer has aggressive biologic characteristics, the postoperative prognosis is poor. Therefore, the impact of PNSP on postoperative outcomes might not be relatively significant in this type of malignancy. In patients with biologically aggressive cancer, it is difficult to rule out the possibility that removing cancer through surgery affects postoperative patient nutritional status and QOL more than preoperative nutritional support. However, there is no denying that many factors of nutrition and QOL of malnourished patients with pancreatobiliary cancer have improved since PNSP and shown comparable clinical outcomes compared with those of well-nourished patients in this study.

The results of this study can be evidence of the clinical effect of preoperative nutritional support in patients with pancreatobiliary cancer. It is also meaningful because this study shows the non-inferiority in postoperative outcomes and QOL of malnourished patients with PNSP after major operation for pancreatobiliary cancer compared to well-nourished patients.

In conclusion, providing preoperative nutritional support to malnourished patients with pancreatobiliary cancer could help improve nutritional status and clinical outcomes. Additionally, a more effective PNSP protocol must be established for patients who do not experience improvement in nutritional status after receiving preoperative nutritional support.

## Supplementary Information

**Additional file 1.**

**Additional file 2.**

**Additional file 3.**

## Data Availability

All data generated or analyzed during this study are included in this published article and its supplementary information files.

## Abbreviations

*LOS*: Length of stay
*QOL*: Quality of life
*PG-SGA*: Patient-Generated Subjective Global Assessment
*PNSP*: Preoperative Nutritional Support Program
*ESPEN*: European Society for Clinical Nutrition and Metabolism
*BMI*: Body mass index
*CBC*: Complete blood count
*CRP*: C-reactive protein
*ECOG*: Eastern Cooperative Oncology Group
*EORTC QLQ-C30*: European Organization for Research and Treatment of Cancer Quality of Life Questionnaire version 3.0
*EN*: Enteral nutrition
*FBS*: Fasting blood sugar
*PN*: Parenteral nutrition
*POD*: Postoperative day
*ASA*: American Society of Anesthesiologists
*HbA1c*: Hemoglobin A1c
*CA 19-9*: Carbohydrate Antigen 19-9
*CEA*: Carcinoembryonic antigen
*LN*: Lymph node
*PD*: Pancreatoduodenectomy
*PPPD*: Pylorus preserving pancreatoduodenectomy
*HPD*: Hepaticopancreatoduodenectomy
*BDR*: Bile duct resection
