# Supplementary material for: Effect of preoperative nutritional support in malnourished patients with pancreatobiliary cancer: a quasi-experimental study
Source: BMC Nutr. 2022 Jul 11;8:61. doi: 10.1186/s40795-022-00555-2 (PMC9277960; doi:10.1186/s40795-022-00555-2)
Supplement: Supplementary file 1 — Additional file 1. [file 40795_2022_555_MOESM1_ESM.docx]

Supplemental Digital Centent

Table S1. Nutritional information of oral supplement (Encover^®^, JW Pharmaceutical Co., Ltd., Korea)

| Ingredient | Content | Ingredient | Content |
| --- | --- | --- | --- |
| Ascorbic Acid | 56.2mg | Biotin | 7.72㎍ |
| Calcium | 88mg | Carbohydrate | 31.24g |
| Chloride | 234mg | Cholecalciferol | 27.2IU |
| Copper | 250㎍ | Cyanocobalamin | 0.64㎍ |
| Fat | 4.46g | Folic Acid | 75㎍ |
| Iron | 1250㎍ | Magnesium | 38.6mg |
| Manganese | 266㎍ | Nicotinamide | 5mg |
| Pantothenic Acid | 1916㎍ | Phosphorus | 88mg |
| Potassium | 276mg | Protein | 8.76g |
| Pyridoxine | 750㎍ | Retinol | 414IU |
| Riboflavin | 490㎍ | Sodium | 147.6mg |
| Thiamine | 760㎍ | Tacopherol | 1300㎍ |
| Vitamin K | 21㎍ | Zinc | 1280㎍ |
| Serving size 200ml, Calories 200kcal | | | |

Table S2. Baseline characteristics of the two subgroups according to improvement in PG-SGA score and grade after the preoperative nutritional support program.

| Factors |  | B/C -> B/C (n = 23) | B/C -> A (n = 22) | *p* |
| --- | --- | --- | --- | --- |
| Age | >75 years | 3 (13.0%) | 4 (18.2%) | 0.634 |
| Sex | Male | 17 (73.9%) | 13 (59.1%) | 0.353 |
|  | Female | 6 (26.1%) | 9 (40.9%) |  |
| ECOG | 0 | 14 (60.9%) | 14 (63.6%) | >0.999 |
|  | 1 | 9 (39.1%) | 8 (36.4%) |  |
| Diagnosis | Bile duct cancer | 13 (56.5%) | 9 (40.9%) | 0.415 |
|  | Gallbladder cancer | 1 (4.3%) | 0 |  |
|  | Pancreatic cancer | 5 (21.7%) | 9 (40.9%) |  |
|  | Others | 4 (17.4%) | 4 (18.2%) |  |
| Type of surgery | PD/PPPD | 18 (78.3%) | 12 (54.5%) | 0.197 |
|  | HPD | 2 (8.7%) | 2 (9.1%) |  |
|  | Hepatectomy + BDR | 3 (13.0%) | 5 (22.7%) |  |
|  | Distal pancreatectomy | 0 | 3 (13.6%) |  |
| *ECOG*, Eastern Cooperative Oncology Group; *PD*, pancreatoduodenectomy; *PPPD*, pylorus preserving pancreatoduodenectomy; *HPD*, hepaticopancreatoduodenectomy; *BDR*, bile duct resection | | | | |

Table S3. Characteristics of the patients who initially presented PG-SGA grade B or C according to preoperative nutritional support outcomes

|  |  | B/C -> B/C  (n = 23) | B/C -> A  (n = 22) | *p* |
| --- | --- | --- | --- | --- |
| Patient factors |  |  |  |  |
| Age | >75 years | 3 (13.0%) | 4 (18.2%) | 0.634 |
| Sex | Male | 17 (73.9%) | 13 (59.1%) | 0.353 |
|  | Female | 6 (26.1%) | 9 (40.9%) |  |
| ECOG | 0 | 9 (39.1%) | 16 (72.7%) | 0.063 |
|  | 1 | 13 (56.5%) | 6 (27.3%) |  |
|  | 2 | 1 (4.3%) | 0 |  |
| ASA score | 1 | 3 (13.0%) | 1 (4.5%) | 0.555 |
|  | 2 | 18 (78.3%) | 18 (81.8%) |  |
|  | 3 | 2 (8.7%) | 3 (13.6%) |  |
| Diagnosis | Bile duct cancer | 13 (56.5%) | 9 (40.9%) | 0.415 |
|  | Gallbladder cancer | 1 (4.3%) | 0 |  |
|  | Pancreatic cancer | 5 (21.7%) | 9 (40.9%) |  |
|  | Others | 4 (17.4%) | 4 (18.2%) |  |
| Nutritional support |  |  |  |  |
| Duration of support |  | 6 (4-17) | 6 (5-35) | 0.646 |
| Type of Support | Oral | 5 (21.7) | 6 (27.3) | 0.857 |
|  | Oral supplement | 10 (43.5) | 10 (45.5) |  |
|  | IV supplement | 8 (34.8) | 6 (27.3) |  |
| Total days of IV support |  | 1 (0-17) | 0 (0-4) | 0.102 |
| Initial day of IV support |  | 1 (1-2) | 2 (1-7) | 0.087 |
| kcal/kg/day |  | 31.7 (20.3-44.5) | 32.6 (24.6-41.9) | 0.114 |
| kcal/kg/day success day (%) |  | 94 (20~100) | 100 (71-100) | 0.051 |
| Protein intake (g/kg/day) |  | 1.2 (0.8-1.6) | 1.4 (0.9-2.0) | 0.005 |
| Protein success day (%) |  | 69 (20-100) | 100 (60-100) | 0.001 |
| Lipid intake (g/kg/day) |  | 0.9 (0.3-1.4) | 1.0 (0.6-1.7) | 0.248 |
| Lipid success day (%) |  | 63 (0-100) | 71 (0-100) | 0.518 |
| Nutritional indices after support |  |  |  |  |
| BMI | < 18.5 kg/m2 | 2 (8.7%) | 0 | 0.157 |
| Anemia | M<13.0; F<12.0 g/dL | 21 (91.3%) | 15 (68.2%) | 0.053 |
| Albumin level | < 3 g/dL | 1 (4.3%) | 0 | 0.323 |
| Protein level | < 6 g/dL | 5 (21.7%) | 3 (13.6%) | 0.477 |
| Cholesterol level | > 200 mg/dL | 4 (17.4%) | 3 (13.6%) | 0.728 |
| Prealbumin level | < 16 mg/dL | 3 (13.0%) | 1 (4.5%) | 0.317 |
| Transferrin level | < 170 mg/dL | 5 (21.7%) | 2 (9.1%) | 0.242 |
| *ECOG*, Eastern Cooperative Oncology Group; *ASA*, american society of anesthesiologists; *BMI*, body mass index | | | | |

Table S4. Analysis of nutritional status at postoperative day 14 and 2 week after discharge. Subgroup analysis according to whether nutritional status improved

| *p*  (vs B/C -> A) ^†^ | | | | | | Postoperative day 14 | 0.491 | 0.302 |  |  | 0.284 | 0.062 | 0.034^#^ | 0.338 | 0.575 | 0.585^#^ |
| --- | --- | --- | --- | --- | --- | --- | --- | --- | --- | --- | --- | --- | --- | --- | --- | --- |
|  |  | |  | | (vs B/C -> B/C) ^†^ |  | 0.202 | 0.076 |  |  | 0.215 | 0.055 | 0.948 | 0.315 | 0.865 | 0.006^#^ |
| Well-nourished (n = 45) | | | | n (%) | |  | 1 (2.2%) | 11 (24.4%) | 33 (73.3%) | 1 (2.2%) | 42 (93.3%) | 7 (15.6%) | 29 (64.4%) | 2 (4.4%) | 37 (84.1%) | 21 (47.7%) |
| PNSP (n = 43) | | *p*^‡^ | | | |  | 0.157 | 0.539 |  |  | 0.059 | 0.003 | 0.121^#^ |  | 0.720 (85.7%) | 0.049 |
|  |  | B/C -> A (n=22) | | n (%) | |  | 0 | 3 (14.3%) | 16 (76.2%) | 2 (9.5%) | 17 (85.0%) | 0 | 7 (35.0%) | 0 | 17 (89.5%) | 11 (57.9%) |
|  |  | B/C -> B/C (n=21) | |  |  |  | 2 (9.1%) | 1 (4.5%) | 19 (86.4%) | 2 (9.1%) | 22 (100%) | 8 (36.4%) | 14 (63.6%) | 0 | 18 (85.7%) | 18 (85.7%) |
|  | |  | | Variables | |  | BMI < 18.5 kg/m^2^ | ECOG 0 | 1 | 2 | Anemia | Albumin < 3 g/dL | Protein < 6 g/dL | Cholesterol > 200 mg/dL | Prealbumin < 16 mg/dL | Transferrin <170 mg/dL |

| Two weeks after discharge | 0.483 | 0.470 |  |  | 0.669 |  | 0.164 | 0.781 | 0.283 | 0.116 | 0.842 |  |  | 0.548 | *PNSP*, preoperative nutritional support program; *ECOG,* Eastern Cooperative Oncology Group; *PG-SGA*, Patient-Generated Subjective Global Assessment  #: Fisher’s exact test, †: Wilcoxon rank sum test |
| --- | --- | --- | --- | --- | --- | --- | --- | --- | --- | --- | --- | --- | --- | --- | --- |
|  | 0.572 | 0.289 |  |  | 0.573^#^ |  | 0.511 | 0.715 | 0.691 | 0.102 | 0.315 |  |  | 0.200 |  |
|  | 4 (9.1%) | 20 (45.5%) | 21 (47.7%) | 3 (6.8%) | 30 (69.8%) | 0 | 4 (9.1%) | 3 (6.8%) | 12 (27.3%) | 5 (11.4%) | 16 (38.1%) | 25 (59.5%) | 1 (2.4%) | 9 (1-22) |  |
|  | 0.900 | 0.346 |  |  | 0.863 |  | 0.335 | 0.945 | 0.524 | 0.012 | 0.726 |  |  | 0.549^†^ |  |
|  | 3 (15.0%) | 9 (45.0%) | 11 (55.0%) | 0 | 15 (75.0%) | 0 | 0 | 1 (5.0%) | 3 (15.0%) | 0 | 7 (36.8%) | 11 (57.9%) | 1 (5.3%) | 10 (3-21) |  |
|  | 3 (13.6%) | 6 (27.3%) | 15 (68.2%) | 1 (4.5%) | 17 (77.3%) | 0 | 1 (4.5%) | 1 (4.5%) | 5 (22.7%) | 6 (27.3%) | 5 (27.8%) | 11 (61.1%) | 2 (11.1%) | 11 (1-20) |  |
|  | BMI < 18.5 kg/m^2^ | ECOG 0 | 1 | 2 | Anemia | Albumin < 3 g/dL | Protein < 6 g/dL | Cholesterol > 200 mg/dL | Prealbumin < 16 mg/dL | Transferrin < 170 mg/dL | PG-SGA grade A | B | C | PG-SGA score |  |
